# Inequalities in the burden of disease due to dementia, including Alzheimer disease, in British Columbia, Canada, from 2001 to 2022

**DOI:** 10.24095/hpcdp.45.10.02

**Published:** 2025-10

**Authors:** Andrea D. Olmstead, Fernanda Ewerling, Shengjie Zhang, Bonnie Henry, Xibiao Ye

**Affiliations:** 1 Office of the Provincial Health Officer, Ministry of Health, Government of British Columbia, Victoria, British Columbia, Canada; 2 Ministry of Health, Government of British Columbia, Victoria, British Columbia, Canada; 3 School of Population and Public Health, University of British Columbia, Vancouver, British Columbia, Canada; 4 School of Health Information Science, University of Victoria, Victoria, British Columbia, Canada

**Keywords:** dementia, burden of disease, disability-adjusted life-years, material and social deprivation, socioeconomic status, mortality, population health, administrative health data

## Abstract

**Introduction:**

Disability-adjusted life-years (DALYs) integrate mortality and prevalence (or incidence) data. DALYs can be used as a surveillance measure to assess dementia burden and inequalities.

**Methods:**

We utilized dementia case and mortality counts from linked administrative data to estimate incidence, prevalence, cause-specific mortality and DALYs in people aged 65 years and older, from 2001 to 2022, in British Columbia, Canada. Dementia-specific mortality rates adjusted for changes in death certification practices over time were estimated using logistic regression that incorporated multiple cause-of-death data from vital statistics records. All measures were stratified by sex; DALYs were also stratified by age and area-based socioeconomic status (SES) quintiles. Average annual percent change (AAPC) in rates was estimated using joinpoint regression.

**Results:**

Age-standardized dementia incidence and prevalence have declined since 2013, while mortality has increased by, on average, 1.6% per year since 2001 (95% CI: 1.4% to 1.8%). Age-standardized DALYs have increased by, on average, 1.4% per year (95% CI: 1.3% to 1.4%). DALY rates are highest in females aged 90 years and older but are increasing more rapidly in males. DALYs have declined for those in the least deprived SES quintile (AAPC: −0.6%; 95% CI: −1.0% to −0.3%) and conversely, have increased—with recent rates the highest—in the most deprived quintile (AAPC: 2.9%; 95% CI: 2.5% to 3.2%).

**Conclusion:**

The socioeconomic gap in dementia disease burden has widened over time in British Columbia. DALYs are highest in females aged 90 years and older, but the overall gap between males and females has declined.

**Highlights**

- Age-standardized disability-adjusted life-years (DALYs) have increased, on average, by 1.4% per year from 2001 to 2022.
- DALY rates by age group are highest in females aged 90 years and older, but they have been increasing over time at a faster rate for males in this age group.
- Dementia DALYs have declined for people living in the least socioeconomically deprived areas (average change of −0.6% per year) but increased in the most deprived areas (average change of 2.9% per year).
- Dementia-specific mortality rates from before 2015 may have been underestimated, contributing to an inflated upward trend from 2001 to 2022.
- After adjusting for underlying causeof- death certification improvements, age-standardized mortality due to dementia has increased by, on average, 1.6% per year from 2001 to 2022.

## Introduction

Dementia refers to a set of symptoms associated with progressive deterioration of cognitive functions caused by neurodegenerative and vascular diseases or injuries that affect daily living.^1^ Alzheimer disease is the most common type of dementia,^2^ making up approximately 60% to 70% of cases in Canada. Based on administrative health data, the Canadian Chronic Disease Surveillance System (CCDSS) identified 499 905 cases of dementia in people aged 65 years and older in Canada in the 2023 to 2024 fiscal year.^3^ The Alzheimer Society of Canada estimates that the total number of cases will reach one million by 2030.^4^

Dementia risk increases with age and is higher for females;3 associations between dementia incidence, prevalence and mortality and socioeconomic factors such as education, income, housing, employment, food security, stress and racial discrimination have also been reported.^5^-^13^

Continuous monitoring and evaluation of dementia inequalities in Canada is necessary to provide insight into the groups that are most affected and at risk, guide appropriate action and evaluate progress resulting from public health activities.^1^ Population-level health administrative datasets contain information that can be used to track disease prevalence and mortality and associations with a few health determinants such as age, sex and geographical region.

The disability-adjusted life-years (DALYs) metric is a comprehensive surveillance measure, adopted by the 2019 Global Burden of Diseases, Injuries, and Risk Factors Study (GBD 2019),^14^ that provides an integrated picture of the impact of disease prevalence and mortality on a population. DALYs are a direct sum of the number of healthy life-years lost due to illness (years lived with disability, or YLDs) and premature death (years of life lost, or YLLs). DALYs are comparable across diseases and allow for monitoring changes in population health and comparing the health of different populations. Data derived from the GBD 2019 demonstrate that globally, from 1990 to 2019, crude incidence and prevalence rates of dementia increased by 148% and 161%, respectively.^14^,^15^ When rates were standardized by age, DALYs increased by, on average, 0.15% per year over the same period.^14^,^15^

The goal of this study was to estimate the dementia disease burden over time in the province of British Columbia, Canada, and to evaluate inequalities using population-level health administrative data combined with methods developed by the GBD 2019. Because of uncertainties in the accuracy of dementia mortality reporting over time, our investigation integrated a methodology to adjust local dementia mortality rates based on multiple cause-of-death (MCOD) data recorded on individual cause-of-death records.^16^ Dementia health inequalities were evaluated in different age groups, in males and in females, and in those living in areas with different socioeconomic status (SES).

## Methods

***Ethics approval***

This study was conducted as part of a population health research program approved by the University of British Columbia Research Ethics Board (Ethics REB #H22-01818) on 25 August 2022.

***Dementia incidence, prevalence and mortality***

Incidence and prevalence counts of dementia, including Alzheimer disease, in people aged 65 years and older were obtained from the British Columbia Chronic Disease Registry (BCCDR) produced by the Office of the Provincial Health Officer of British Columbia. The BCCDR tracks incidence and prevalence of 25 chronic conditions using predefined case definitions applied to administrative health databases, including practitioner visits (Medical Service Plan), hospitalizations (Discharge Abstract Database) and prescription dispensation records (PharmaNet). BCCDR case ascertainment methods are derived from algorithms developed and validated by CCDSS and incorporate British Columbia–specific criteria into the Canada-based algorithms.^3^,^17^ The BCCDR identifies cases of dementia in people with one or more hospitalizations with a dementia code (International Classification of Diseases [ICD]-10 codes G30 and F00–F03 or ICD-9 codes 046.1, 290, 294.1, 294.2, 331.0, 331.1, 331.5 and 331.82); three or more medical visits with a dementia ICD code at least 30 days apart within 2 years; or one or more dementia drug dispensation records (donepezil, rivastigmine, galantamine or memantine). Incidence includes the number of new cases identified within a fiscal year, while prevalence is the total number of cases of dementia identified any time before the end of the fiscal year of interest in anyone aged 65 years and older and living in British Columbia at that time. Dementia deaths were counted from the British Columbia Vital Statistics Agency death registry where the underlying cause of death (UCOD) on the death certificate is attributed to dementia as defined by the GBD 2019 (ICD-10 codes: F00–F03, G30–G31.1, G31.8–G31.9; ICD-9 codes: 290–290.9, 294.1–294.9, 331–331.2) for selected individuals aged 65 years and older.

***Adjusted dementia mortality rates***

A recent report suggests that trends in increasing age-standardized dementia mortality rates (i.e. deaths with dementia as the UCOD) in countries such as Australia and the United States may not be accurate.^16^ Changes in death certification and coding practices (i.e. describing the order, type and association of events that resulted in a person’s death) and increasing awareness of dementia as a UCOD may have inflated the upward trend in mortality rates over the past two decades.^18^-^21^ Adair et al. developed a regression model that incorporates MCOD data recorded on the death certificates of people with dementia to estimate the probability that dementia was the true UCOD.^16^ We applied this methodology to estimate adjusted mortality rates in British Columbia and used these in calculating DALYs.

Briefly, all MCODs were extracted from vital events records from 1 January 2000 to 31 December 2022 for anyone aged 65 years and older who had dementia recorded on Part 1 or Part 2 of their death record. These MCODs were categorized into 17 cause-of-death variables (e.g. stroke, diabetes, cancer, injuries, etc.), as described by Adair et al.^16^ MCODs that did not fall into these categories (about 13% of the entries) were not included. More than 60% of these unused codes were “garbage codes” (i.e. could not be official causes of death) as defined by the GBD 2019. Separate models were fit for males and females using logistic regression, where the dependent variable was dementia as the UCOD (yes or no) and the independent variables included the 17 MCODs, age (continuous), death year and death place type (home, hospital, non-hospital care facility or other). The resulting coefficients were then used to calculate the probability that dementia was the UCOD for each individual based on the available data. The coefficient for 2019 was used for the death year, because this year is hypothesized to have the most accurate death certification practices for dementia, assuming these practices are improving over time and that there were disruptions to determining cause of death during the first 2 years of the COVID-19 pandemic. Individual probabilities of dementia as the UCOD were summed to obtain yearly adjusted dementia mortality counts.

***Disability-adjusted life-years***

DALYs were calculated as the sum of YLLs and YLDs for dementia in a given population, time and sex using the following equation:

DALY_c,s,a,t,q_ = YLL_c,s,a,t,q_ + YLD_c,s,a,t,q_

where *c* stands for cause (dementia); *s* for sex (male, female, total); *a* for age (65+ years, by 5-year age group); *t* for time (by fiscal year, from 1 April 2001 to 31 March 2022); and *q* for area-based SES quintiles (detailed in the subsection, “Area-based SES”).

YLLs were calculated using the following equation: 

YLL_c,s,a,t,q_ = N_c,s,a,t,q_ L_a_

where *N* stands for number of deaths and *L* for the gap between age of death and optimal life expectancy. Optimal life expectancy values were obtained from the GBD 2019 theoretical minimum risk life table.^22^ This reference table was constructed based on the lowest observed age-specific mortality rates by location and sex from all locations with populations of more than five million in 2016.

YLDs were calculated using the following equation:

YLD_c,s,a,t,q_ = ∑ [P_c,s,a,t,q_ DW_c,s,a_ SP]

where *P* stands for prevalence counts, *DW* for disability weight and *SP* for severity proportion. The severity proportion is the proportion of individuals in the population estimated to be experiencing mild, moderate or severe dementia.^23^^,p.966^ GBD 2019 used a systematic review to collect information on the proportion of individuals in each dementia severity class, with information largely based on data from three population surveys in Australia and the United States. The Clinical Dementia Rating scale was used as the reference definition for severity classification, along with a doctor-given diagnosis, according to the *Diagnostic and Statistical Manual of Mental Disorders* (third, fourth or fifth edition) or ICD case definitions, as their reference definition for dementia. (For further details, refer to GBD 2019 Supplementary Appendix 1^23^^,p.964^.)

The severity proportion is paired with the disability weight (mild, moderate or severe) to calculate overall YLDs. Disability weights were obtained from the GBD 2019^23^^,p.1547^ and are further described by Salomon et al.^24^ Disability weights are measured on a scale of 0 to 1 (where 0 equals a state of full health and 1 equals death) and represent the magnitude of health loss associated with a specific health status.

***Area-based SES***

Since socioeconomic factors are difficult to obtain from administrative datasets, DALYs were stratified according to the material and social deprivation index (MSDI) developed by the Institut national de sant publique du Qubec.^25^ The MSDI is used to monitor social inequalitiesin health. Deprivation scores (material deprivation based on income, education and employment; social deprivation based on marital status, lone parent status and living alone) are assigned to small area units (grouping between 400 and 700 persons) from the Canadian census called dissemination areas (DA). DAs are relatively homogeneous in terms of socioeconomic conditions and are linkable to postal codes found in administrative databases. However, some DAs are excluded from the MSDI because of low population numbers, collective households or other factors. Many individuals with dementia live in facilities where DA-level census data are suppressed; that is, 34% of prevalent dementia cases in the BCCDR from fiscal year 2016 to 2017 lived in a DA with no deprivation score. 

To overcome this limitation, we first imputed missing deprivation scores by classifying the corresponding DAs as urban or rural (i.e. lying within or outside, respectively, a census metropolitan area or census agglomeration) using Statistics Canada’s Geographic Attribute file.^26^ For DAs with missing scores, the smallest geographical area (census tract < census subdivision < census division) with available deprivation scores and with the same urban/rural assignment was identified and the median value of the scores within that region were assigned to that DA.

With each census cycle (2001, 2006, 2011 and 2016), DA boundaries changed (substantial changes in 2001 relative to 2006, and minor changes in 2011 and 2016). New deprivation scores were calculated and assigned to those DAs. In our dataset, each person was linked to a 2016 DA, but because our dataset contained cases going back to 2001, we used MSDI scores calculated over time and imputed a score for each fiscal year. We did this by geographically aligning the 2016 DAs with DAs from previous cycles using the R package tongfen (R Foundation for Statistical Computing, Vienna, AT), which facilitates joining disparate spatial boundary data together into a common geographical region. First, tongfen was run with all four census cycles to join the 2001 to 2016 DA data. This resulted in a file that underwent substantial aggregation into new hybrid-DAs that are relatively large compared to regular DAs. Tongfen was run a second time using only 2006 to 2016 DA data, which underwent less aggregation, largely maintaining original DA sizes. The two datasets were joined, keeping only the 2001 to 2006 portion from the first step and the 2006 to 2016 portion from the second step, and each region was assigned an associated 2016 DA identification number.

Annual scores were derived from the 5-year MSDI scores using linear interpolation, that is, three separate linear functions were fit between each pair of proximate census years to allow estimating the scores for the intermediate years.

Finally, we combined deprivation index quintiles from the separate material and social deprivation quintiles (using the second suggested grouping method described by Azevedo Da Silva et al.^27^^,p.6^).

***Data analysis and statistical methods***

All analyses were performed in R version 4.2.2. Rates were calculated by dividing surveillance measure counts by mid-fiscal year population counts and reported per 100 000 population by fiscal year (1 April to 31 March, inclusive). Canadian 2011 Census population estimates were used as the standard population for age-standardization. Rates were stratified by sex (all measures) and by 5-year age group and SES quintiles (DALYs only).

We calculated uncertainty intervals (UIs) for YLLs, YLDs and DALYs using bootstrapping, sampling 5000 draws at each step of the calculations. Point estimates were calculated as the 50th percentile of the sampling draws, the lower UI as the 2.5th percentile and the upper UI as the 97.5th percentile.

We calculated average annual percent change (AAPC) for discontinuous trends over time using joinpoint regression with the segmented package version 1.6-0 in R.28 AAPC for continuous upwards or downwards trends where no significant breakpoints were detected were derived from the slope using a log–linear regression model with the following equation: 

AAPC = 100נ(exponent(slope) − 1).

## Results

***Age-standardized dementia incidence, prevalence and mortality over time, by sex***

Dementia incidence in people aged 65years and older declined, changing by an average of −0.4% (95% confidence interval [CI]: −0.7% to −0.2%) per year over the study period, with similar trends observed for males and females (Figure 1A; Table 1). Age-standardized prevalence rose by, on average, 5.5% (95% CI: 4.6% to 6.4%) per year from 2002 to 2004 and 2.0% (95% CI: 1.9% to 2.2%) per year from 2005 to 2013. The age-standardized prevalence has since declined, changing by −0.7% (95% CI: −0.9 to −0.6%) per year (Figure 1A; Table 1).

**Figure 1A:**
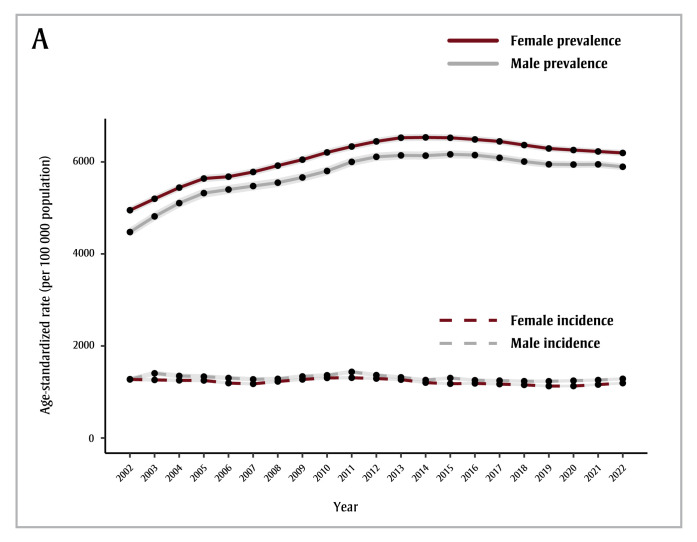
Age-standardized dementia incidence and prevalence, by fiscal year and sex, individuals aged 65+ years, British Columbia, Canada

**Notes:** Years displayed on the x-axes denote the end of that fiscal year, e.g. 2002 stands for the 2001/2002 fiscal year. The light grey shaded areas represent the 95% confidence intervals. 

**Table 1:** APCs for individual segments and AAPC for the entire trend for age-standardized dementia incidence, prevalence, unadjusted and adjusted mortality, YLDs, YLLs and DALYs, total and by sex, individuals aged 65+ years, British Columbia, Canada

Group	Trend 1		Trend 2		Trend 3		AAPC, %
	Years^a^	APC, %^b^	Years	APC, %^b^	Years	APC, %^b^	
Incidence^c^							
Total	N/A	N/A	N/A	N/A	N/A	N/A	−0.4 (−0.7 to −0.2)
Male	N/A	N/A	N/A	N/A	N/A	N/A	−0.4 (−0.6 to −0.1)
Female	N/A	N/A	N/A	N/A	N/A	N/A	−0.5 (−0.7 to −0.2)
Prevalence							
Total	2002–2004	5.5 (4.6 to 6.4)	2005–2013	2.0 (1.9 to 2.2)	2014–2022	−0.7 (−0.9 to −0.6)	1.2 (1.1 to 1.3)
Male	2002–2004	6.8 (5.5 to 8.0)	2005–2013	2.0 (1.8 to 2.2)	2014–2022	−0.6 (−0.8 to −0.4)	1.4 (1.3 to 1.5)
Female	2002–2004	4.8 (4.1 to 5.6)	2005–2013	2.0 (1.9 to 2.1)	2014–2022	−0.7 (−0.9 to −0.6)	1.1 (1.1 to 1.2)
Mortality^c^							
Total	N/A	N/A	N/A	N/A	N/A	N/A	3.3 (2.9 to 3.7)
Male	N/A	N/A	N/A	N/A	N/A	N/A	3.7 (3.3 to 4.2)
Female	N/A	N/A	N/A	N/A	N/A	N/A	3.1 (2.7 to 3.5)
Adjusted mortality							
Total	2002–2010	0.7 (0.0 to 1.4)	2011–2022	2.3 (1.9 to 2.7)	N/A	N/A	1.6 (1.4 to 1.8)
Male	2002–2014	1.4 (0.7 to 2.1)	2015–2022	2.7 (1.9 to 3.6)	N/A	N/A	1.9 (1.6 to 2.2)
Female	2002–2010	0.3 (−0.5 to 1.2)	2011–2022	2.2 (1.9 to 2.6)	N/A	N/A	1.5 (1.2 to 1.7)
YLDs							
Total	2002–2004	5.4 (4.8 to 6.0)	2005–2013	2.0 (1.9 to 2.1)	2013–2022	−0.8 (−0.9 to −0.7)	1.2 (1.1 to 1.2)
Male	2002–2004	6.8 (5.5 to 8.1)	2005–2013	2.0 (1.8 to 2.2)	2013–2022	−0.6 (−0.8 to −0.4)	1.4 (1.3 to 1.5)
Female	2002–2004	4.8 (4.0 to 5.6)	2005–2013	2.0 (1.9 to 2.2)	2013–2022	−0.8 (−0.9 to −0.7)	1.1 (1.1 to 1.2)
YLLs							
Total	2002–2011	0.5 (0.1 to 0.9)	2012–2022	2.3 (2.0 to 2.6)	N/A	N/A	1.5 (1.3 to 1.6)
Male	2002–2013	1.1 (0.4 to 1.7)	2014–2022	2.8 (1.9 to 3.7)	N/A	N/A	1.8 (1.5 to 2.0)
Female	2002–2010	0.3 (−0.4 to 0.9)	2011–2022	2.1 (1.7 to 2.5)	N/A	N/A	1.4 (1.2 to 1.6)
DALYs^c^							
Total	N/A	N/A	N/A	N/A	N/A	N/A	1.4 (1.3 to 1.4)
Male	N/A	N/A	N/A	N/A	N/A	N/A	1.5 (1.3 to 1.7)
Female	N/A	N/A	N/A	N/A	N/A	N/A	1.3 (1.2 to 1.4)

**Abbreviations:** AAPC, average annual percent change; APC, annual percent change; DALYs, disability−adjusted life−years; YLDs, years lived with disability; YLLs, years of life lost. 

^a^ The year aligning with end of fiscal year period is denoted in the table (e.g. 2002 = 2001/2002 fiscal year). 

^b^ APC for each breakpoint is presented separately (if breakpoints were detected). 

^c^ No significant breakpoints were detected for incidence, mortality and DALY rate trends. 

We estimated dementia mortality rates adjusted for changes in certification practices as higher than unadjusted rates across all years except 2022 (Figure 1B). Age-standardized dementia mortality has been trending upwards over the past two decades (Figure 1A); an AAPC of 3.3% (95% CI: 2.9% to 3.7%) was estimated using unadjusted rates while an AAPC of 1.6% (95% CI: 1.4% to 1.8%) was estimated using adjusted mortality rates. Of note, unadjusted mortality estimates show a decline in the rate of dementia mortality in the first year of the COVID-19 pandemic (March 2020 to March 2021) compared to the previous year, while adjusted mortality rates predicted an increase in dementia deaths in the first year of the pandemic.

**Figure 1B:**
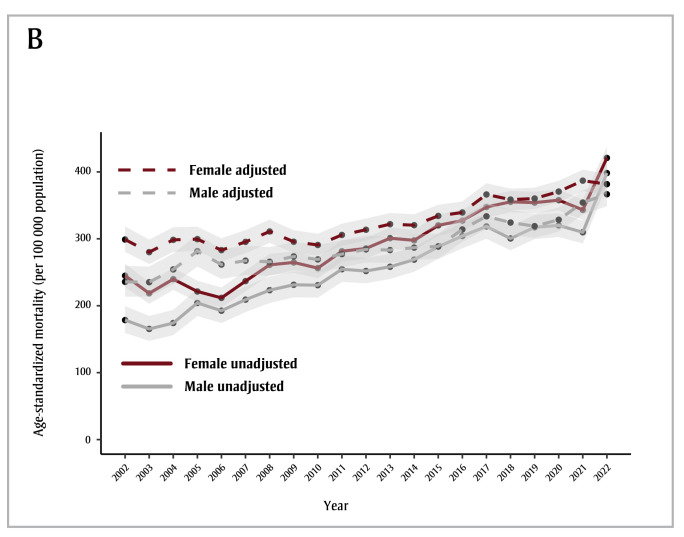
Age-standardized dementia mortality, unadjusted and adjusted to take into account changes in death certification practices, by fiscal year and sex, individuals aged 65+ years, British Columbia, Canada

**Notes: **Years displayed on the x-axes denote the end of that fiscal year, e.g. 2002 stands for the 2001/2002 fiscal year. The light grey shaded areas represent the 95% confidence intervals. 

***Age-standardized YLDs, YLLs and DALYs due to dementia over time, by sex and age***

**Figure 2:**
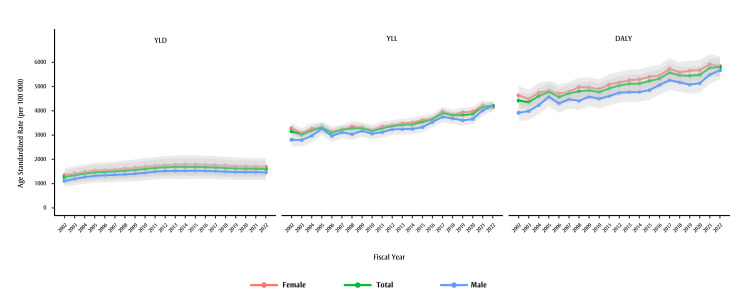
Age-standardized YLDs, YLLs and DALYs due to dementia, by fiscal year and by sex, individuals aged 65+ years, British Columbia, Canada

**Abbreviations:** YLDs, years lived with disability; YLLs, years of life lost; DALYs, disability-adjusted life-years.

**Notes:** Years displayed on the x-axes denote the end of that fiscal year, e.g. 2002 stands for the 2001/2002 fiscal year.

YLD trends for dementia mirror prevalence and declined, changing by −0.8% (95% CI: −0.9 to −0.7) per year since 2013 (Figure 2). The AAPC for YLLs computed using adjusted mortality is 1.5% (95% CI: 1.3% to 1.6%). The AAPC for DALYs is 1.4% (95% CI: 1.3% to 1.4%). Similar trends were observed for males and females over most of the study period, but the AAPC is higher in males for all three measures. Also of note, YLLs and DALYs declined for females (−1.8% and −1.4%, respectively) and increased for males (4.8% and 3.3%, respectively) in 2022 compared to 2021, resulting in a narrowing gap for these measures.

The light grey shaded areas represent the uncertainty intervals calculated using bootstrapping (2.5th and 97.5th percentiles from 5000 draws).

***Age-specific DALYs due to dementia over time, by 5-year age group and sex***

**Figure 3:**
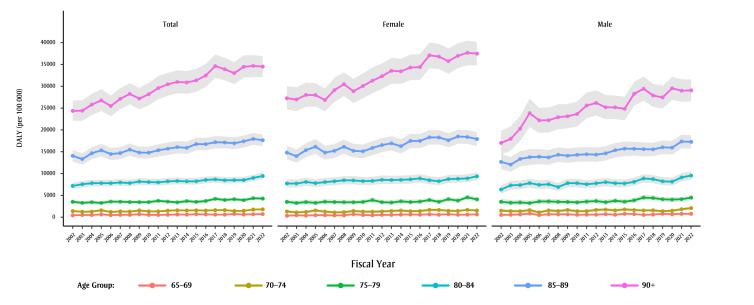
Age-standardized YLDs, YLLs and DALYs due to dementia, by fiscal year and by sex, individuals aged 65+ years, British Columbia, Canada

**Abbreviation: **DALYs, disability-adjusted life-years.

**Notes: **Years displayed on the x-axes denote the end of that fiscal year, e.g. 2002 stands for the 2001/2002 fiscal year. 

Figure 3 shows that the burden of disease due to dementia increases with age, as expected, and is higher in females than males in the highest age group, 90 years and older. Moreover, the greatest increase in burden over time is also occurring in the highest age group, with an AAPC of 1.8% (95% CI: 1.6% to 2.0%). Although DALY rates are higher in females aged 90 years and older than males in this age group, AAPC of DALY in males is significantly higher than in females (male AAPC: 2.9%, 95% CI: 2.4% to 3.5%; female AAPC: 1.9%, 95% CI: 1.7% to 2.1%).

The light grey shaded areas represent the uncertainty intervals calculated using bootstrapping (2.5th and 97.5th percentiles from 5000 draws). 

***Age-standardized DALYs due to dementia over time, by SES quintile and sex***

**Figure 4:**
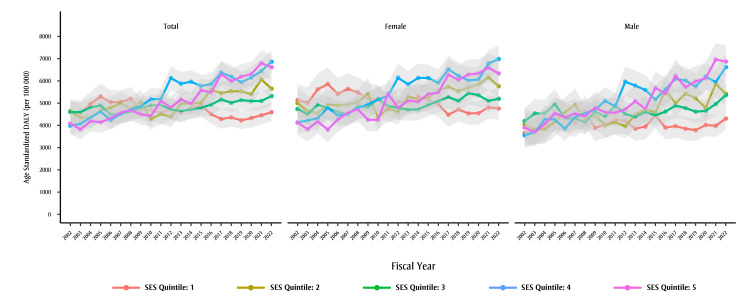
Age-standardized DALYs due to dementia, by fiscal year, by sex and by SES quintilea, individuals aged 65+ years, British Columbia, Canada

**Abbreviations: **DALYs, disability-adjusted life-years; SES, socioeconomic status.

**Notes: **Years displayed on x-axes denote the end of that fiscal year, e.g. 2002 stands for the 2001/2002 fiscal year. 

DALYs stratified by SES quintile show a marked difference between those in the most deprived (quintile 5) versus the least deprived (quintile 1) regions (Table 2; Figure 4). Age-standardized DALYs dropped, changing by −0.6% (95% CI: −1.0% to −0.3%) per year in the least deprived quintile while they increased by 2.9% (95% CI: 2.5% to 3.2%) per year in the most deprived quintile. AAPCs in males and females were similar in the most deprived quintile (male AAPC: 2.9%, 95% CI: 2.5% to 3.3%; female AAPC: 2.8%, 95% CI: 2.4% to 3.3%). However, in the least deprived quintile, we observed a decline among females (AAPC: −0.9%, 95% CI: −1.3 to −0.5%), while rates did not change significantly over time among males (AAPC: 0%, 95% CI: −0.5 to 0.4%).

**Table 2:** AAPC in age-standardized DALYs due to dementia, stratified by SES quintile and sex, individuals aged 65+ years, British Columbia, Canada, fiscal years 2001/2002 to 2021/2022

SES quintile	AAPC^a^, % (95% CI)		
	Total	Male	Female
1 (least deprived)	−0.6 (−1.0 to −0.3)	0 (−0.5 to 0.4)	−0.9 (−1.3 to −0.5)
2	1.4 (1.0 to 1.8)	1.7 (1.2 to 2.3)	1.3 (0.8 to 1.7)
3	0.6 (0.4 to 0.8)	0.6 (0.2 to 1.0)	0.7 (0.4 to 0.9)
4	2.9 (2.4 to 3.3)^b^	2.8 (2.2 to 3.3)^b^	2.5 (2.1 to 2.9)
5 (most deprived)	2.9 (2.5 to 3.2)	2.9 (2.5 to 3.3)	2.8 (2.4 to 3.3)

**Abbreviations:** AAPC, average annual percent change; CI, confidence interval; DALYs, disability-adjusted life-years; SES, socioeconomic status. 

^a^ AAPC was calculated from log–linear regression slope as the trends had no significant breakpoints, except where otherwise indicated.

^b^ This AAPC was calculated from joinpoint regression due to two significant breakpoints in the trendline. 

The light grey shaded areas represent the uncertainty intervals calculated using bootstrapping (2.5th and 97.5th percentiles from 5000 draws).

^a^ SES quintile 1 refers to the least deprived and SES quintile 5 the most deprived.

## Discussion

In this study we investigated the disease burden of dementia over 20 years in British Columbia and found declining incidence and prevalence and increasing mortality. The overall age-adjusted burden (DALY) is also increasing over time, with AAPCs highest in males, those aged 90 years and older and those living in regions with low SES.

Our modelling of adjusted dementia mortality rates supports the hypothesis that dementia as a UCOD may have been underreported in British Columbia, especially before 2015, which resulted in underestimated cause-specific mortality rates. This is reflected in the higher mortality rates modelled in earlier years, when MCOD data are used to adjust dementia mortality counts, compared to the number of deaths reported on vital statistics records. Still, even after accounting for improvements in UCOD certification practices, we measured an AAPC of 1.6% in age-adjusted dementia mortality, which is comparable to the change reported by Adair et al. for Australia and the United States.^16^ This increase may reflect (1) changing MCOD reporting practices over time that were not taken into account by our model, and (2) a shift to dementia being a cause of death because other, previously more common causes (e.g. cardiovascular disease–related deaths) have declined.^16^,^29^ These findings reiterate the need for caution when comparing dementia mortality rates derived from vital statistics over several years and that, whenever possible, modelling approaches should be used to account for changes over time.

DALY data from our study show that, on average, the disease burden of dementia in British Columbia has increased by 1.4% per year. The GBD 2019 estimated that the dementia DALY rate per 100000 population in Canada was similar in 2001 (309.07; 95% UI: 144.94–656.73) and 2019 (310.66; 95% UI: 145.73–648.29).^30^ The difference in our DALY measurements are likely due to mortality estimates; the GBD 2019 estimated similar mortality rates in 2001 and 2019 in Canada,^30^ which differs from our estimated 36% increase in dementia mortality from 2001 to 2022.

Stratification by age showed that DALYs are highest and increasing at the greatest rate among people aged 90 years and older. The higher AAPC in those aged 90 years and older is a combination of increasing prevalence before 2012 and rising mortality over most of the study period in this age group (data not shown). This may reflect a decline in deaths due to other causes and an increasing likelihood of dementia being the UCOD, but may also reflect a true increase in dementia burden in some populations. DALY rates in this age group have consistently been higher in females than in males over time, while in other age groups the DALY rates are similar in males and females. This suggests that the higher overall burden in females aged 90 years and older may be a consequence of their longer average lifespan. This burden may also be influenced by the underdiagnosis of dementia in younger people and those with milder stages of the disease, and that the disease is less likely to contribute to or be recognized as a cause of death in younger people.^18^,^19^

The differences in trends observed across SES quintiles are particularly important and highlight a widening socioeconomic gap in health outcomes.^11^,^31^-^33^ SES comprises multiple factors that affect people’s ability to engage in health activities, afford medical care and housing, and manage stress.^25^,^34^-^36^ Lower SES is consistently associated with worse health outcomes, reflecting disparities in access to care, health literacy and other social determinants of health.^31^,^32^,^36^,^37^ Many of the modifiable factors that influence risk of dementia are more prevalent in people with lower SES; these include diabetes, hypertension, smoking, alcohol consumption, depression, poor diet (i.e. resulting from food insecurity or barriers to and shifts away from traditional and cultural food consumption) and less formal education.^33^-^35^,^38^,^39^ People in higher SES groups often have better access to cutting-edge diagnostic tools, novel medications and specialized care40,41 that can lead to earlier detection, more effective management of dementia and improved survival rates. Reviewing the literature for this report highlighted the paucity of recent disaggregated data on dementia in Canada; such data are needed to inform policy and direct resources to the most at-risk populations.^38^,^42^ Enhanced surveillance is thus needed to drive evidence-informed policies that build tailored health and social services and empower communities to improve health in meaningful and enduring ways.^34^,^42^

***Strengths and limitations***

Strengths of this study are that it utilized population-level health administrative datasets linkable to demographic information, including area-based SES, that allowed us to evaluate local disease burden and inequalities. Another strength was that we used a validated case definition to identify cases of dementia in British Columbia.^17^

A limitation of using this dataset is the impact of the COVID-19 pandemic on health care use, that likely influenced the reported rates for the fiscal years 2020 to 2021 and 2021 to 2022. Changes in trends observed during pandemic years should be interpreted with caution.^43^ Dementia was reported on36% of COVID-19death certificates issued in Canada from January 2020 to February 2021, higher than any other comorbidity.^44^ This is likely due to a combination of factors, including the overlapping and enhanced risk for serious COVID-19 illness associated with living with dementia, at older age and in a long-term care facility early in the pandemic.^45^-^49^ Vital statistics records in our dataset predicted a decline in dementia deaths, while adjusted rates predicted an increase, in the first pandemic year (March 2020 to March 2021). There are two possible interpretations: (1) people who would have likely otherwise died from dementia, as predicted by MCOD modelling, died as a result of COVID-19 infection; or (2) difficulties in determining the true cause of death of people with dementia who contracted COVID-19 near their time of death may have resulted in inaccurate reporting of dementia mortality.^50^,^51^

Other limitations arising from the secondary use of administrative health data include potential misclassification biases, with mild cases of dementia likely underrepresented, and the incorrect assignment of some individuals as having dementia (i.e. due to medical coding errors or misdiagnoses). Although dementia mortality rates were adjusted for changes over time, death misclassifications likely persist in our dataset. In addition, the methodology used to compute DALYs relied on metrics developed by the GBD 2019, such as the optimal life expectancy table, disability weights and severity proportions; these may differ from equivalent metrics in Canada.

## Conclusion

In this study we provided a methodological framework for surveillance of the disease burden of dementia in Canada. Our results underscore the importance of considering medical coding and death certification practices and socioeconomic factors in the interpretation of chronic disease statistics and highlight demographics that should be the focus of enhanced prevention and care in British Columbia.

## Acknowledgements

We would like to thank Tim Adair (University of Melbourne, Melbourne, AU) for providing guidance on the use of the multiple cause-of-death regression analysis; Anders Erickson (British Columbia Ministry of Health) for the material and social deprivation index (MSDI) quintile imputation; Alyssa J. Parker and Richard Mercer (both with the Office of the Provincial Health Officer of the British Columbia Ministry of Health) for revising the MSDI imputation methodology; Promit Ananyo Chakraborty (Office of the Provincial Health Officer of the British Columbia Ministry of Health) for contributing to the review of the literature for this manuscript; and Larry Shaver and Catherine Pelletier (both with the Public Health Agency of Canada) for study review and feedback. 

We acknowledge the contributions of all staff involved in the collection, integration and management of the administrative data used in this analysis, including team members in the Office of the Provincial Health Officer of the British Columbia Ministry of Health (Henry Ngo, Kayla McLean, Yao Nie and Ioana Sevcenco) and the residents of British Columbia whose data were used for this investigation.

## Funding

This work was supported via the Enhanced Dementia Surveillance Initiative of the Public Health Agency of Canada (Contract 6D023-203002).

## Conflicts of interest

The authors have no conflicts of interest to declare.

## Authors’ contributions and statement

ADO: Conceptualization, formal analysis, investigation, methodology, project administration, visualization, writing—original draft, writing—review and editing.

FE: Conceptualization, methodology, project administration, writing—review and editing.

SZ: Data curation, formal analysis, writing—review and editing.

BH: Conceptualization, supervision, writing—review and editing.

XY: Conceptualization, methodology, supervision, writing—review and editing.

The content and views expressed in this article are those of the authors and do not necessarily reflect those of the Government of Canada or the British Columbia Government.

This study was carried out without the involvement of the Government and is not meant to express their views or opinions.

## Data availability statement

The individual level data used in this study were obtained from the British Columbia Ministry of Health Data Warehouse called Healthideas, which is not publicly accessible to protect individual privacy and confidentiality. Access to deidentified data may be obtained through one of the following platforms: Population Data BC (https://www.popdata.bc.ca/); the Data Innovation Program (https://www2.gov.bc.ca/gov/content/data/finding-and-sharing/data-innovation-program); or the Health Data Platform BC (https://healthdataplatformbc.ca/). The names of all the datasets used are in the methods section. Names of the variables used in this study may be requested by contacting the corresponding author.
